# Heat treatment and false-positive heartworm antigen testing in ex vivo parasites and dogs naturally infected by *Dirofilaria repens* and *Angiostrongylus vasorum*

**DOI:** 10.1186/s13071-017-2444-6

**Published:** 2017-11-09

**Authors:** Luigi Venco, Simone Manzocchi, Marco Genchi, Laura H. Kramer

**Affiliations:** 1 Clinica Veterinaria Lago Maggiore, 28041 Arona, Italy; 2 Private practitioner, Pavia, Italy; 3 Department of Veterinary Sciences, University of Parma, Parma, Italy

**Keywords:** *Dirofilaria* spp., Antigen tests, Cross-reactivity

## Abstract

**Background:**

Heartworm antigen testing is considered sensitive and specific. Currently available tests are reported as detecting a glycoprotein found predominantly in the reproductive tract of the female worm and can reach specificity close to 100%. Main concerns regard sensitivity in the case of light infections, the presence of immature females or cases of all-male infections. Research and development have been aimed at increasing sensitivity. Recently, heat treatment of serum prior to antigen testing has been shown to result in an increase in positive antigen test results, presumably due to disruption of natural antigen–antibody complexes. Cross-reactions in dogs with both natural and experimental infections with *Angiostrongylus vasorum* and *Spirocerca lupi* have been reported, but cross-reactions with other helminths have not been extensively studied. In order to evaluate potential cross-reactivity with other canine and feline parasites, two studies were performed. Study 1: Live adults of *Dirofilaria immitis*, *Dirofilaria repens*, *Toxocara canis*, *Toxocara cati*, *Dipylidium caninum*, *Taenia taeniaeformis* and *Mesocestoides* spp. larvae were washed and incubated in tubes with saline solution. All worms were alive at the time of removal from the saline. Saline solutions containing excretory/secretory antigens were then tested for heartworm with six different, commercially available antigen tests. All results were evaluated blind by three of the authors. Study 2: Sera from dogs with natural infections by *A. vasorum* or *D. repens*, living in areas free of heartworm disease, were tested with the same tests before and after heat treatment (103 °C for 10 min).

**Results:**

Results suggest that antigens detected by currently available tests are not specific for *D. immitis.* They may give positive results through detection of different parasites’ antigens that are normally not released into the bloodstream or released in a low amount and/or bound to antibodies. Tests may even detect antigens released by male *D. immitis* adult worms. *D. repens* appears to release more detectable antigens than the other worms studied.

**Conclusions:**

Cross-reaction with *A. vasorum* and *D. repens* does occur in the field and could potentially occur with other helminths. Heat treatment decreases specificity by enhancing cross-reactivity.

## Background

Heartworm antigen (Ag) testing is perceived as being very accurate, with a specificity virtually approaching 100%. There are, however, concerns regarding sensitivity in cases of light, immature female, or all-male infections, as the sensitivity of heartworm antigen tests depends on the worm burden, and the sex and age of the parasites [1–3]. Currently available tests are reported as detecting a glycoprotein found predominantly in the reproductive tract of the female worm, and efforts have been made to increase test sensitivity. However, potential cross-reactions in dogs with both natural and experimental infections with *A. vasorum* and *S. lupi* have been recently reported in dogs [4, 5], while others have reported cross-reactivity with *Acantocheilonema reconditum* in dogs and *A. odendhali* in sea lions [6, 7].

Despite reports of currently available tests showing specificity ranging from 84.5% to 100%, with most of them ranging between 97% and 100% [8], the potential concern about possible cross-reactions with other helminths has not been extensively evaluated, due to the assumption that most of the tests are based on monoclonal antibodies against *Dirofilaria immitis* Ags. Recently, heat treatment of serum prior to antigen testing has been shown to result in an increase in positive antigen test results [9–11], presumably due to disruption of natural antigen-antibody complexes. A recent field study [12] has suggested that increased sensitivity with heat treatment could decrease specificity allowing possible cross-reactions with *D. repens*. A lack of specificity due to cross- reactivity could strongly affect the positive predictive value of testing in different epidemiological areas or scenarios [13], leading to unnecessary or inappropriate treatments.

Here, two different studies (experimental and field) were performed in order to evaluate potential cross-reactivity of currently available antigen tests, with several different canine and feline parasites.

## Methods

### Study 1

Live adult helminths were collected from privately owned dogs and cats with natural parasitic infections. Eleven adults of *D. immitis* were obtained during therapeutic surgical removal of heartworms, with the Ishihara technique [14]. Three worms were from a dog treated with doxycycline (10 mg/kg once a day for one month) and ivermectin (8 μg/kg every 2 weeks for three treatments) before surgical removal. Fourteen adult *D. repens* were collected from subcutaneous nodules during therapeutic surgical removal with a mini-invasive technique [15]. Three adult *Toxocora canis*, four adult *Toxocara cati,* five *Toxocara* spp. larvae, two adult *Dipylidium caninum* and one *Taenia taeniformis* were collected from dogs and cats immediately following owner-requested euthanasia and necropsy for unrelated diseases. Finally, *Mesocestoides* sp. larvae were obtained during routine abdominal surgery.

All parasites were individually identified based on morphology, washed carefully with saline solution and incubated for 30 min at room temperature (25 °C) in a tube (a single worm for each tube) with 3 mL of saline solution. All worms were alive at the time of removal from the saline. The saline solutions containing excretory/secretory antigens were diluted (1:8) and then tested with: SNAP® HTWM (IDEXX Laboratories, Westbrook, Maine, USA), SNAP® 4Dx® Plus (IDEXX Laboratories, Westbrook, Maine, USA), Witness® Dirofilaria (Zoetis, Rome, Italy), Speed Diro™ (Virbac, Milan, Italy), PetChek® HTWM PF (IDEXX, Westbrook, Maine, USA) and DiroCHEK® Heartworm Antigen Test (Synbiotics, San Diego, California, USA). Three tubes with normal saline solution were included in the study as negative controls. All results were recorded by three, blinded authors (LK, SM, MG) and scored according to the scheme reported in Table 1.

**Table 1:** Assessment scores for the tests used in the study

Score	Witness®	SNAP® 4Dx® Plus	SNAP® HTWM	Speed Diro™	PetChek® HTWM PF Optical Reading NEG 0.0420+ 0.05 POS 0.6529	DiroCHEK® Optical Reading NEG 0.0562 POS 0.3754
0	No reaction	No reaction	No reaction	No reaction	≥ 0.0920	≥ 0.0562
+	Mild PR	Mild PR	1 spot less bright than control spot	1-3 into the Speed reading chart^a^	≥ 0.0921 ≤ 0.37245	≥ 0.0563 ≤ 0.2158
++	Clear PR but less than control band/spot	Clear PR but less than control spot	1 spot as bright as control spot	4-7 into the Speed reading chart^a^	≥ 0.37245 ≤ 0.6529	≥ 0.2159 ≤ 0.3754
+++	PR as clear as control band/spot	PR as clear as control band/spot	2 spots	8-10 into the Speed reading chart^a^	≥0.6530	≥ 0.3755

^a^Color chart provided by manufacturer and used to measure the intensity of the color change

### Study 2

Sera from privately owned dogs with natural *A. vasorum* or *D. repens* infections, living in areas free of heartworm disease, were tested before and after heat treatment (103 °C for 10 min) as described [9] with the same tests used in Study 1.

The three dogs naturally infected by *A. vasorum* were positive for serum antigens using IDEXX Angio Detect™ and for larvae using the Baermann fecal examination technique. All were on regular heartworm chemoprophylaxis (oral ivermectin, 12 months a year). Dogs ranged in age from 4 months to 2 years old.

The four dogs naturally infected by *D. repens* were all microfilaremic as evaluated by a modified Knott’s test, and all presented with subcutaneous nodules. For negative controls, sera from two dogs and one cat with no parasites visible on necropsy were used.

## Results

Study 1 evaluated different heartworm antigen tests on saline solutions containing excretory/secretory antigens of *D. immitis*, *D. repens* and several intestinal helminths. Saline solutions from all adult *D. immitis*, both male and female, were positive on all tests except for one male worm that resulted negative on SNAP 4Dx® Plus and Speed Diro™ (Table 2). In the case of immature heartworms, only the immature female was positive and only on three of the six tests used (Witness®, Speed Diro® and DiroCHEK®), while the immature male was negative on all (Table 3). One adult male and one adult female from a dog that had been treated with a combination of doxycycline and ivermectin were positive on all tests (except for SNAP^®^ 4Dx® Plus that was negative for the female) with, however, weaker reactions (Table 3). Table 4 reports the results of testing of saline solutions containing antigens from *D. repens*. All tests were positive for both male and female adult worms, with few exceptions. Table 5 shows results from testing of solutions containing antigens from a variety of intestinal helminths. Interestingly, all cestodes except *Mesocestoides* spp. gave positive results on all tests. DiroCHEK® and Witness were often positive for antigens from adult *T. canis* and *T. cati*. The *T. canis* and *T. cati* females were positive on virtually all tests. All negative controls resulted negative on all tests.

**Table 2:** Results of tests on saline solutions containing excretory/secretory antigens of adult *Dirofilaria immitis*

Dog Code	Worm Gender	SNAP® HTWM	SNAP® 4Dx® Plus	Witness®	Speed Diro™	PetChek®	DiroCHEK®
C	F	+	+	+	+	+	+
C	F	+	+	++	+	+	++
C	F	+++	++	+++	+++	+++	+++
K	F	+++	+++	+++	+++	+++	+++
K	M	+	0	+	0	+	+
K	M	++	++	++	++	++	++

**Table 3:** Results of tests on saline solutions containing excretory/secretory antigens of immature and adult *Dirofilaria immitis* from a dog pretreated with doxycycline and ivermectin

Dog Code	Worm Gender	SNAP® HTWM	SNAP® 4Dx® Plus	Witness®	Speed Diro™	PetChek®	DiroCHEK®
Young immature adults							
KL	F	0	0	+	+	0	+
KL	M	0	0	0	0	0	0
Dog treated with doxycycline: 10 mg/kg SID for 1 month and ivermectin 8 μg/kg every 2 weeks for 3 Tx							
M	F	+	0	++	+	+	+
M	M	+	+	+	+	+	+
M	M	0	0	+	0	0	+

**Table 4:** Results of tests on saline solutions containing excretory/secretory antigens of *Dirofilaria repens* adults

Dog Code	Worm Gender	SNAP® HTWM	SNAP® 4Dx® Plus	Witness®	Speed Diro™	PetChek®	DiroCHEK®
GN	F	++	+	++	++	++	+++
GN	F	+++	+	+++	++	+++	+++
GN	F	+	0	++	+	++	++
GN	F	+++	+++	+++	+++	+++	+++
GN	F	++	++	++	++	+++	+++
GN	F	++	+	+++	++	+++	+++
GN	F	+	+	++	++	++	+++
GN	F	++	+	++	+	++	++
GN	F	+	+	++	+	+	++
GN	M	+	0	++	+	+	+
GN	M	0	0	+	+	+	++
GN	M	+	0	+	+	0	+
G	F	+++	+++	+++	+++	+++	+++
C	F	+	+	+	++	++	+++

**Table 5:** Results of tests on saline solutions containing excretory/secretory antigens of *Toxocara canis, Toxocara cati, Dipylidium caninum, Taenia taeniaeformis* and *Mesocestoides* sp.

Parasite	Worm Gender	SNAP® HTWM	SNAP® 4Dx® Plus	Witness®	Speed Diro™	PetChek®	DiroCHEK®
*T. canis*	F	+	+	+	+	++	++
*T. canis*	M	0	0	+	0	0	0
*T. canis*	M	0	0	0	0	0	0
*T. cati*	F	++	0	++	+	++	++
*T. cati*	F	+	0	++	+	++	++
*T. cati*	M	0	0	++	+	0	++
*T. cati*	M	0	0	++	0	0	++
*T. cati*	Larvae^a^	0	0	0	0	0	0
*T. taeniaeformis* ^b^		++	+++	++	+	++	++
*D. caninum* ^c^		+	++	+	+	+	+
*D. caninum* ^c^		+	++	+	++	++	+
*Mesocestoides* sp.^d^		0	0	0	0	0	0

^a^Five short immature adult/larvae (recovered from the intestinal lumen) in the same tube. ^b^Whole parasite. ^c^One terminal gravid segment for each tube. ^d^Five tetrathyridium (peritoneal larvae) into the same tube

Study 2 was aimed at identifying potential cross-reactivity in dogs naturally infected with *A. caninum* and *D. repens* and the eventual increase in cross-reactions following heat treatment of serum. Serum samples from dogs with natural *A. vasorum* infection gave conflicting results on heartworm antigen testing before heat treatment (Table 6). SNAP HTWM® and PetChek® yielded positive results in 3/3 dogs, Witness® in 2/3 and DiroCHEK® in 1/3. SNAP® 4Dx® Plus and Speed Diro™ were negative for all samples. After heat treatment, SNAP HTWM® and PetChek® still yielded positive results in all samples, but with a more intense positive reaction. The previously negative sample tested with Witness® became positive, while the two previously negative with DiroCHEK® became positive. Finally, Speed Diro™ became positive for one of the two previously negative samples, while SNAP^®^ 4DX® became positive for all previously negative samples. All tests were negative in *D. repens-* infected dogs before heat treatment, except for Witness® and DiroCHEK®, which detected low antigen levels in two of the dogs (Table 7). After heat treatment, all of the tests gave positive results.

**Table 6:** Results of serological testing before and after heat treatment in *Angiostrongylus vasorum* naturally infected dogs

Dog Code	Before/ After Heat Tx	SNAP® HTWM	SNAP® 4Dx® Plus	Witness®	Speed Diro™	PetChek®	DiroCHEK®
TS	B	+	0	+	0	+	+
TS	A	++	+	++	+	++	++
IG	B	+	0	+	0	++	0
IG	A	++	+	++	0	++	+
NO^a^	B	+	0	0	0	+	0
NO^a^	A	++	+	+	0	++	+
C.D1^b^	B	0	0	0	0	0	0
C.D2^b^	A	0	0	0	0	0	0
C.C1^b^	B	0	0	0	0	0	0
C.D1^b^	A	0	0	0	0	0	0
C.D1^c^	B	0	0	0	0	0	0
C.D1^c^	A	0	0	0	0	0	0

B = before heat treatment. A = after heat treatment. ^a^4-month-old dog. ^b^Negative control dogs. ^c^Negative control cat

**Table 7:** Results of serological testing before and after heat treatment in *Dirofilaria repens-* naturally infected dogs

Dog Code	Before/ After Heat Tx	SNAP® HTWM	SNAP® 4Dx® Plus	Witness®	Speed Diro™	PetChek®	DiroCHEK®
G	B	0	0	+	0	0	+
G	A	++	+	++	+	++	++
GN	B	0	0	0	0	0	0
GN	A	++	+	++	++	++	+++
LU	B	0	0	0	0	0	0
LU	A	+	+	+	+	+	+
BR	B	0	0	+	0	0	+
BR	A	++	+	+++	+	+	+++

*B* before heat treatment. *A* after heat treatment

All of the challenged tests in the study correctly identified negative controls (sera from dogs and cats without parasites on necropsy) before and after heat treatment.

## Discussion

Results of Study 1 suggest that antigens detected by currently available tests are not specific for *D. immitis.* They could therefore give positive results through detection of other parasites’ antigens that are normally not released into the bloodstream (or released in low amounts and bound to antibodies). Tests were able to detect Ags released by male *D. immitis* adult worms (in vitro), suggesting that even all-male infections could be diagnosed with the highly sensitive tests actually on the market, contrary to what is currently thought. Immature *D. immitis* and those from dogs treated with doxycycline and ivermectin gave weaker positive results, in agreement with published data [16], which suggests that doxycycline and ivermectin treatment could lead to false-positive results.

*Dirofilaria repens* appears to release more detectable antigens than the other worms studied in vitro*.* Results from Study 2 suggest that *D. repens* infection can lead to false-positive results on heartworm antigen testing, as published and confirmed for *A. vasorum*. Furthermore, heat treatment of the serum sample appears to cause an increase in cross-reactions. This should be taken into account in those areas, for example, many European countries, that are endemic for heartworm and where *D. repens* is common and spreading. Increasing test sensitivity by heat treatment decreases specificity and should not be recommended for routine use, at least in areas where *D. repens and A. vasorum* are present.

This could also be true for other parasites, considering the results of Study 1. However, results of the present study need to be confirmed by further in vivo studies according to previous research [17]. Indeed, it is likely that several of these parasites do not normally release antigens into the bloodstream. There may be, however, particular conditions (ie, enteritis) where parasite antigens do begin to circulate. Heat treatment may further increase the detection of these antigens and is probably not to be recommended in any situation.

## Conclusions

Lack of specificity as a consequence of cross-reactivity could strongly affect the positive predictive value of testing in different epidemiological areas or scenarios leading to unnecessary or inappropriate treatment. Major efforts should be made to increase specificity, rather than sensitivity, of heartworm antigen tests, particularly in countries where *D. repens* and *A. vasorum* are found. Furthermore, in an asymptomatic dog, a *D. immitis-*positive antigen test that is not supported by other evidence of disease (thoracic radiographs, echocardiography) or by the presence of circulating microfilariae, should be carefully interpreted, particularly in low endemic areas. In these cases, other parasitic diseases should be ruled out before starting treatment.

## Abbreviation

Ag: heartworm antigen

## References

[1] Lee AC, Bowman DD, Lucio-Forster A, Beall MJ, Liotta JL, Dillon R (2011). Evaluation of a new in-clinic method for the detection of canine heartworm antigen. Vet Parasitol.

[2] Atkins CE (2003). Comparison of results of three commercial heartworm antigen test kits in dogs with low heartworm burdens. J Am Vet Med Assoc.

[3] Courtney CH, Zeng Q (2001). Comparison of heartworm antigen test kit performance in dogs having low heartworm burdens. Vet Parasitol.

[4] Schnyder M, Deplazes P (2012). Cross-reactions of sera from dogs infected with *Angiostrongylus vasorum* in commercially available *Dirofilaria immitis* test kits. Parasit Vectors.

[5] Aroch I, Rojas A, Slon P, Lavy E, Segev G, Baneth G (2015). Serological cross-reactivity of three commercial in-house immunoassays for detection of *Dirofilaria immitis* antigens with *Spirocerca lupi* in dogs with benign esophageal spirocercosis. Vet Parasitol.

[6] Gillis JM, Smith RD, Todd KS (1984). Diagnostic criteria for an enzyme-linked immunosorbent assay for occult heartworm disease: standardization of the test system in naturally exposed dogs. Am J Vet Res.

[7] Krucik DD, Van Bonn W, Johnson SP. Association between positive canine heartworm (*Dirofilaria immitis)* antigen results and presence of *Acanthocheilonema odendhali* microfilaria in California sea lions (*Zalophus californianus*). J Zoo Wildl Med 2016;47(1):25-28. 10.1638/2014-0116.1 27010261

[8] Rohrbach BW, Patton S (2013). Effects of diagnostic test accuracy and treatment efficacy on the occurrence of suspected failure of heartworm prophylaxis in dogs. J Vet Intern Med.

[9] Little SE, Munzing C, Heise SR, Allen KE, Starkey LA, Johnson EM (2014). Pre-treatment with heat facilitates detection of antigen of *Dirofilaria immitis* in canine samples. Vet Parasitol.

[10] Little SE, Raymond MR, Thomas JE, Gruntmeir J, Hostetler JA, Meinkoth JH (2014). Heat treatment prior to testing allows detection of antigen of *Dirofilaria immitis* in feline serum. Parasit Vectors.

[11] Velasquez L, Blagburn BL, Duncan-Decoq R, Johnson EM, Allen KE, Meinkoth J (2014). Increased prevalence of *Dirofilaria immitis* antigen in canine samples after heat treatment. Vet Parasitol.

[12] Ciucă L, Genchi M, Kramer L, Mangia C, Miron LD, Prete LD (2016). Heat treatment of serum samples from stray dogs naturally exposed to *Dirofilaria immitis* and *Dirofilaria repens* in Romania. Vet Parasitol.

[13] Erb HN (2011). Prior probability (the pretest best guess) affects predictive values of diagnostic tests. Vet Clin Pathol.

[14] Arita N, Yamane I, Takemura N (2003). Comparison of canine heartworm removal rates using flexible alligator forceps guided by transesophageal echocardiography and fluoroscopy. J Vet Med Sci.

[15] Venco L, Valenti V, Bertazzolo W, Genchi M, Grandi G (2011). A mini-invasive procedure for removal of adult *Dirofilaria repens* from subcutaneous nodules in dogs. Intern J Appl Res Vet Med.

[16] Bazzocchi C, Mortarino M, Grandi G, Kramer LH, Genchi C, Bandi C (2008). Combined ivermectin and doxycycline treatment has microfilaricidal and adulticidal activity against *Dirofilaria immitis* in experimentally infected dogs. Int J Parasitol.

[17] Song KH, Hayasaki M, Cho KW, Lee SE, Kim DH (2002). Cross-reactivity between sera from dogs experimentally infected with *Dirofilaria immitis* and crude extract of *Toxocara canis*. Korean J Parasitol.

